# Increased GFAP concentrations in the cerebrospinal fluid of patients with unipolar depression

**DOI:** 10.1038/s41398-021-01423-6

**Published:** 2021-05-21

**Authors:** Maike Michel, Bernd L. Fiebich, Hanna Kuzior, Sophie Meixensberger, Benjamin Berger, Simon Maier, Kathrin Nickel, Kimon Runge, Dominik Denzel, Benjamin Pankratz, Miriam A. Schiele, Katharina Domschke, Ludger Tebartz van Elst, Dominique Endres

**Affiliations:** 1 Department of Psychiatry and Psychotherapy, Medical Center—University of Freiburg, Faculty of Medicine, University of Freiburg, 79104 Freiburg, Germany; 2 Clinic of Neurology and Neurophysiology, Medical Center—University of Freiburg, Faculty of Medicine, University of Freiburg, 79106 Freiburg, Germany; 3 Center for Basics in Neuromodulation, Faculty of Medicine, University of Freiburg, 79106 Freiburg, Germany

**Keywords:** Depression, Predictive markers

## Abstract

Inflammatory processes involving altered microglial activity may play a relevant role in the pathophysiology of depressive disorders. Glial fibrillary acidic protein (GFAP) and calcium-binding protein S100B are considered microglial markers. To date, their role has been studied in the serum and tissue material of patients with unipolar depression but not in the cerebrospinal fluid (CSF). Therefore, the aim of the current study was to examine GFAP and S100B levels in the CSF of patients with major depression to better understand their role in affective disorders. In this retrospective study, 102 patients with unipolar depression and 39 mentally healthy controls with idiopathic intracranial hypertension were investigated. GFAP and S100B levels were measured using commercially available ELISA kits. CSF routine parameters were collected during routine clinical care. The mean values of GFAP and S100B were compared using age (and sex) corrected ANOVAs. Matched subgroups were analyzed by using an independent sample *t*-test. In addition, correlation analyses between GFAP/S100B levels and CSF routine parameters were performed within the patient group. Patients with unipolar depression had significantly higher levels of GFAP than controls (733.22 pg/ml vs. 245.56 pg/ml, *p* < 0.001). These results remained significant in a sub-analysis in which all controls were compared with patients suffering from depression matched 1:1 by age and sex (632.26 pg/ml vs. 245.56 pg/ml, *p* < 0.001). Levels of S100B did not differ significantly between patients and controls (1.06 ng/ml vs. 1.17 ng/ml, *p* = 0.385). GFAP levels correlated positively with albumin quotients (*p* < 0.050), S100B levels correlated positively with white blood cell counts (*p* = 0.001), total protein concentrations (*p* < 0.001), and albumin quotients (*p* = 0.001) in the CSF. The significance of the study is limited by its retrospective and open design, methodological aspects, and the control group with idiopathic intracranial hypertension. In conclusion, higher GFAP levels in patients with depression may be indicative of altered microglia activity, especially in astrocytes, in patients with unipolar depression. In addition, correlation analyses support the idea that S100B levels could be related to the integrity of the blood–brain/CSF barrier. Further multimodal and longitudinal studies are necessary to validate these findings and clarify the underlying biological processes.

## Introduction

With a lifetime prevalence of more than 10%, depression is among the most common mental disorders, thereby imposing a substantial personal, economic, and societal burden^1^. The most common pathophysiological explanation of depression relies on the monoamine hypothesis, which relates depressive symptoms to a lack of neurotransmitters such as serotonin, increased degradation of monoamines, or impaired monoamine sensing^2^. However, in recent years, an increasing number of studies have suggested that inflammatory processes may also be responsible for depressive symptomatology^3–10^. As a result, the role of glial cells and their specific markers in body fluids have been investigated increasingly. The concentrations of glial fibrillary acidic protein (GFAP) in the cerebellum, prefrontal cortex, and anterior cingulate cortex have been found to be lower in patients with depression than in healthy controls^11–13^. In addition, decreased numbers of astrocytes have been found in hippocampal regions of patients with major depressive disorder^14^. Among other functions, GFAP is a crucial factor for an intact blood–brain barrier^15^. Moreover, proteins in the S100 protein family can influence inflammatory processes. They regulate a variety of cell types, such as astrocytes, lymphocytes, and smooth muscle cells^16^. Elevated serum concentrations of S100B have been found in patients with depression and may prove valuable for evaluating both diagnosis and treatment response^17–23^. Most studies that have examined these inflammatory markers in the context of major depression relied on serum or tissue samples. The concentration, origin, and role of inflammatory markers in the cerebrospinal fluid (CSF) of patients with depression have received considerably less attention in psychiatry (for an overview of previous studies see Table 1). However, CSF investigations are needed to better understand the potential role of these biomarkers in the central nervous system, as they can provide detailed insights into intrathecal processes.

**Table 1:** Overview of selected clinical studies investigating GFAP and S100B in cerebrospinal fluid.

Study (by publication year)	Patients/controls	Results related to GFAP and S100
Studies comprising patients with affective disorders		
^61^	9 patients with MDD undergoing ECT	S100B was not changed by clinically successful ECT
^33^	65 elderly women without MDD, 13 with MDD	- GFAP in CSF was not related to depression
		- Women developing dementia within 10 years had increased levels of GFAP in CSF
^35^	133 patients with BD and 86 healthy controls	- No difference of S100B levels in CSF between controls and patients
		- No correlations between S100B in CSF and clinical variables or medication
^34^	31 patients with MDD and 32 healthy controls	Levels of S100B in CSF did not differ between MDD patients and healthy controls
^36^	Japanese cohort of 94 patients with schizophrenia, 68 with BD, 104 with MDD and 118 healthy controls	- S100B in CSF was positively correlated with Hamilton sleep subscale in MDD
		- S100B in CSF was positively correlated with symptom severity in schizophrenia
Studies analyzing patients with different non-affective disorders		
^62^	22 asphyxiated and 8 non-asphyxiated newborns	S100 in CSF correlated positively with indicators for poor prognosis
^63^	12 patients with schizophrenia and 17 controls	- Higher levels of S100B in CSF and serum in the schizophrenia group
		- No differences in GFAP concentrations
^64^	40 consecutive patients with non-inflammatory neurological disorders	S100B in CSF was negatively correlated with severity of depressive symptoms in BDI-II questionnaire
^65^	24 patients with varicella zoster infection of the central nervous system and 14 controls	GFAP levels in CSF were elevated in patients with encephalitis compared to controls
^66^	94 patients with AD and 39 controls	GFAP was not associated with Aβ peptides, but increased with age in controls
^67^	23 patients with PD undergoing deep brain stimulation of the subthalamic nucleus	GFAP concentrations in CSF increased shortly after surgery, but normalized after few months and remained normal over more than a decade afterwards
^68^	49 patients with primary Sjögren’s syndrome	In PCA, S100B dominated a component related to fatigue.
^69^	90 patients with neurological symptoms without neurological diagnosis	GFAP did not indicate signs of neuronal or astroglial damage
^70^	39 patients with NMOSD, 69 with MS, 5 with optic neuritis and 37 controls	GFAP levels in CSF were elevated in patients with NMOSD and antibodies against aquaporin 4 compared to controls

*AD* Alzheimer’s disease, *BD* bipolar disorder, *ECT* electroconvulsive therapy, *MDD* major depressive disorder, *MS* multiple sclerosis, *NMOSD* neuromyelitis optica spectrum disorder, *PCA* principal component analysis, *PD* Parkinson’s disease.

### **Rationale of this study**

The present study aimed to investigate the potential role of the microglial markers GFAP and S100B in the CSF of patients diagnosed with unipolar depression and compare their levels with those of a control group of participants with idiopathic intracranial hypertension (IIH, formerly termed pseudotumor cerebri). Furthermore, the correlative relationship of GFAP/S100B with CSF routine parameters was investigated.

## Participants and methods

The study was part of a larger retrospective project that was approved by the local ethics committee (Faculty of Medicine, University of Freiburg, vote no. EK-Fr 609/14). Lumbar punctures were performed after carefully gathering information and after obtaining written informed consent either as a part of routine clinical care to rule out organic causes of depressive syndromes or—regarding the control group—as a diagnostic investigation or treatment approach for IIH.

### Study sample

In this study, consecutive patients diagnosed with unipolar depression (*N* = 102) were compared to controls with IIH (*N* = 39). Only patients aged from 18 to 65 years were included. Patients diagnosed with unipolar depressive syndromes were treated as inpatients at the Department of Psychiatry and Psychotherapy, University of Freiburg. They were diagnosed by experienced professional psychiatrists based on the criteria in the International Statistical Classification of Diseases and Related Health Problems, 10th revision. Patients diagnosed with schizophrenia, bipolar disorder, or underlying dementia were excluded. Previous neurological illnesses were not a fundamental exclusion criterion, but only patients who received a lumbar puncture during inpatient psychiatric treatment to clarify the depressive syndrome were included. The control group has already been described in previous studies^10,24^. Control subjects were also between 18 and 65 years old, and they additionally had to be free of any psychiatric diagnosis and not have taken psychotropic medication. No matching between patients and controls regarding sex or age was performed for the whole cohort of study participants (for details see Table 2). However, for a sub-analysis, an optimal 1:1 matching (https://cran.r-project.org/web/packages/MatchIt/MatchIt.pdf) by age and sex was implemented using the MatchIt package^25^ in R^26^, which accesses functions from the optmatch package^27^. Thus, the calculations were performed using logistic regression (distances as propensity scores). From 134 study participants (96 patients and 38 controls) with available GFAP findings, 38 optimal matching patients were assigned to the 38 control subjects (total *N* = 76).

**Table 2:** Clinical data of patients and controls.

	Patients (*N* = 102)	Controls (*N* = 39)	Statistics
Sex	47 M: 55 F	6 M: 33 F	***p*** **=** **0.001**
Mean age (age range)	44.25 ± 13.63 (18–65 years)	34.62 ± 12.03 (18–61 years)	***p*** **<** **0.001**
Diagnosis			
Depressive episode with psychotic symptoms	19/102 (19%)	–	
Depressive episode without psychotic symptoms	83/102 (81%)	–	
Severity of depression			
Mild	0/102 (0%)	–	
Moderate	5/102 (5%)	–	
Severe	97/102 (95%)	–	
Course of disease			
Recurrent/chronic	77/102 (75%)	–	
First episode	25/102 (25%)	–	
Neurological comorbidity			
Neurovascular	*n* = 1 (1%)	–	
Traumatic	*n* = 1 (1%)	–	
Polyneuropathy	*n* = 1 (1%)	–	
Migraine/Headache	*n* = 7 (7%)	–	
Restless-leg’s Syndrome	*n* = 3 (3%)	–	
Vertigo	*n* = 2 (2%)	–	
Overall	*n* = 15 (15%)	–	
Psychotropic drugs at the time of sampling			
Medicated	*n* = 99 (97%)	–	
Unmedicated	*n* = 3 (3%)	–	

*CSF* cerebrospinal fluid, *MRI* magnetic resonance imaging, *EEG* electroencephalography, *F* female, *M* male, *SSRI* selective serotonin reuptake inhibitor, *SSNRI* selective serotonin/noradrenaline reuptake inhibitor.

Bold values indicates statistically significant results.

### Cerebrospinal fluid analysis and instrumental diagnostics

The routine CSF analysis included the determination of the following dimensional values (used for correlation analyses): white blood cell (WBC) count, protein concentration, albumin quotient (AQ), and immunoglobulin (Ig)G index. The measuring was performed according to an established methodology^7,8,28^. The routine analyses were carried out in the CSF laboratory, Department of Neurology and Neurophysiology, University of Freiburg (https://www.uniklinik-freiburg.de/neurologie/klinik/diagnostische-einrichtungen/liquor-labor.html). The residual CSF material was then deep-frozen at −80 °C. Only patients and controls with sufficient residual material were included for GFAP and S100B analyses. The mean storage period of the CSF material was 3.60 ± 2.86 years for the patient group and 3.53 ± 2.33 years for the control group. Therefore, the mean storage time did not differ significantly between the groups (*T* = 0.149; *p* = 0.882).

### Measurement of GFAP and S100B

GFAP measurements were performed using commercially available high-sensitivity ELISA kits from Aviscera Bioscience (Santa Clara, USA), and S100B analyses were performed using high-sensitivity ELISA kits from Cloud-Clone Corp. (Houston, TX, USA). The ELISA kits were used in accordance with the manufacturer’s specifications, with the exception of using CSF samples. Both kits have not yet been validated for CSF testing.

### Data handling and statistical analyses

Statistical analyses were performed using Statistical Package for the Social Sciences, version 25 (IBM Corp., Armonk, NY). Age and GFAP levels (only for age and sex matched subgroups) were compared using independent sample *t*-tests, differences in sex ratio were calculated with *χ*^2^. Univariate ANOVAs with age as a covariate were performed for comparison of S100B and GFAP. In a post hoc analysis, we added univariate ANOVAs with age and sex correction for comparison of S100B and GFAP concentrations. Levels of routine CSF parameters (WBC count, total protein, AQ, IgG index) were analyzed using univariate ANOVAs with age and sex correction. S100B and GFAP concentrations were correlated with routine CSF parameters (WBC count, total protein, AQ, IgG index) and different clinical/psychometric scores (suicide attempts, number of earlier inpatient stays, Clinical Global Impression (CGI) score, Global Assessment of Functioning (GAF) score, and psychopathological scores following the German Association for Methodology and Documentation in Psychiatry (AMDP scores)) in the patient group using Pearson correlation coefficients. Significance was set to *p* values of <0.05. Given the exploratory approach, no correction for multiple testing was performed.

## Results

### Sociodemographic and clinical data

Tables 2 and 3 summarize the sociodemographic and clinical data of the study group. The sex distribution differed significantly between patients and controls (*p* = 0.001), with 54% of patients and 85% of controls being female. Likewise, a significant difference in age was found between patients and controls (*p* < 0.001). Most patients (75%) experienced a recurrent or chronic depressive episode, and 25% of patients had experienced their first episode. Five patients had moderate depression (5%) and 97 patients (95%) had severe depression. A total of 99 patients (97%) received psychotropic medication. Neurologic comorbidities occurred in 15% of patients.

**Table 3:** Characterization of the patient group.

	Patients with unipolar depression (*N* = 102)
Marital status	
Single	48 (47%)
Married	36 (35%)
Divorced	12 (12%)
Widowed	5 (5%)
Unknown	1 (1%)
Level of education	
Low	21 (21%)
Middle	20 (20%)
High	55 (54%)
Other	1 (1%)
Unknown	5 (5%)
Work situation	
Unemployed	9 (9%)
Others not working	5 (5%)
Working	53 (52%)
In training	14 (14%)
Retired	18 (18%)
Housewife/-man	3 (3%)
Housing situation	
Alone	43 (42%)
With partner/family	46 (45%)
With parents/guardian	11 (11%)
Other	1 (1%)
Unknown	1 (1%)

### GFAP/S100B findings

The concentrations of GFAP and S100B in CSF are presented in Table 4. In ANOVAs corrected for age, S100B levels did not differ significantly between patients and controls, yet patients with unipolar depression had a significantly higher concentration of GFAP than controls. In a post hoc analysis with additional correction for sex, the results regarding GFAP concentrations remained significant (*F* = 31.057, *p* < 0.001), and S100B levels still showed no significant differences between the groups (*F* = 1.531, *p* = 0.218). When comparing all controls (*N* = 38) with available GFAP results and a matched group of patients with depression (*N* = 38), differences in age (patient group: 36.18 ± 12.97 years; controls: 34.84 ± 12.11 years; *T* = 0.466; *p* = 0.642) and sex (patients: five males and 33 females; controls: six males and 32 females; Chi^2^ = 0.106, *p* = 0.744) were no longer evident. The differences in GFAP levels between patients suffering from depression (632.26 ± 309.18) and controls (245.56 ± 176.25) remained highly significant (*T* = 6.698, *p* < 0.001).

**Table 4:** S100B and GFAP concentrations in both study groups.

	Patients (*N* = 102)	Controls (*N* = 39)	Statistics
GFAP pg/ml (Mean ± SD)	733.22 pg/ml ± 401.42 pg/ml (*N* = 96)^a^	245.56 pg/ml ± 176.25 pg/ml (*N* = 38)^a^	*F* = 41.380
			***p*** **<** **0.001**
S100B ng/ml (Mean ± SD)	1.06 ng/ml ± 0.88 ng/ml	1.17 ng/ml ± 1.08 ng/ml	*F* = 0.759
			*p* = 0.385
	(*N* = 100)^a^		

*SD* standard deviation.

^a^A total of 102 patients and 39 controls were studied. GFAP measurement was successful in 96 of the 102 patients and in 38 of 39 controls. S100B measurement was successful in 100 of the 102 patients.

Bold values indicates statistically significant results.

### Basic CSF findings and instrumental diagnostics

The routine CSF findings are presented in Table 5. The mean total protein concentration and AQ levels were significantly higher in patients than in controls. There were no significant differences in WBC counts and IgG indices.

**Table 5:** Findings in cerebrospinal fluid routine diagnostics.

	Patients (*N* = 102)	Controls (*N* = 39)	Statistics^a^
WBC count/µl (Mean ± SD)	2.08 ± 3.53	2.60 ± 7.59 (*N* = 35)^b^	*p* = 0.700
Total protein in mg/l (Mean ± SD)	462.68 ± 195.84	309.33 ± 142.53	***p*** = **0.007**
Albumin quotient (Mean ± SD)	5.80 ± 2.56	3.93 ± 1.81	***p*** **<** **0.001**
IgG Index in mg/l (Mean ± SD)	0.49 ± 0.08	0.50 ± 0.04	*p* = 0.540

*WBC* white blood cell, *SD* standard deviation, *IgG* immunoglobulin G, *OCBs* oligoclonal bands.

^a^Corrected for age and sex.

^b^One control patient suffered from self-limiting reactive pleocytosis.

Bold values indicates statistically significant results.

### Correlation analyses

S100B levels in the group of patients with unipolar depression correlated positively with WBC count (*r* = 0.323, *p* = 0.001), total protein (*r* = 0.359, *p* < 0.001), and AQs (*r* = 0.327, *p* = 0.001). GFAP levels in the patient group correlated positively with AQs (*r* = 0.201, *p* < 0.050). No significant correlations between S100B or GFAP levels and suicide attempts, number of earlier inpatient stays, CGI, GAF, or AMDP scores were detected.

## Discussion

The main finding of the current study was that the GFAP concentration in the CSF was significantly higher in patients with unipolar depression than in controls with IIH. In addition, a positive correlation between S100B levels and established blood–brain/CSF barrier parameters (AQ and protein concentration) was found in the patient group.

### The present findings in the context of earlier studies

GFAP levels are known to be altered in brain damage—for example, as a result of traumatic injuries or in the course of neurodegenerative or neuroinflammatory processes^29,30^. GFAP may also be involved in long-term potentiation^31^. So far, only few studies have investigated the concentrations of GFAP in the CSF of patients with major depression^32^. In the only other comparable study, GFAP in CSF was not found to be related to major depression in elderly women^33^. However, in contrast to the current study, this was a much older patient group, which could possibly explain the differences to our study results (with higher GFAP levels). As outlined above, several other studies have found lower GFAP levels and decreased numbers of astrocytes in different cerebral regions of patients suffering from depression compared to healthy controls^11–14^. In contrast to the present investigation, these studies utilized postmortem tissue samples instead of CSF and may therefore not be directly comparable as CSF samples exclusively measure extracellular concentrations of GFAP, while in tissue samples, intracellular and membrane-bound proteins add to the signal intensity in concentration measurements. Moreover, secretion of GFAP into CSF with subsequent distribution in the whole CSF spaces may have a diluting effect regarding cerebral tissue and lead to a relative enrichment in CSF. Still, the discrepancy between elevated concentrations of GFAP in CSF and decreased GFAP levels in brain tissue samples needs to be further addressed, ideally in studies combining both analyses for the same patients.

The lack of a significant difference in the concentrations of S100B in CSF between patients and controls in the present study is in agreement with earlier findings in patients with major depression^34^ and bipolar disorder^35^. In a group of Japanese patients with major depression, S100B was positively correlated with the Hamilton sleep subscale^36^, no similar correlation with the AMDP score for sleep dysfunction was detected in the present study. A meta-analysis of inflammatory biomarkers in the CSF of patients with mood disorders found that total protein, albumin, and AQs were higher in patients with mood disorders than in healthy controls^37,38^. The basic CSF findings from the current study indicating increased protein levels and AQs are in line with these previous results.

### Pathophysiological implications of the GFAP and S100B findings

Pathologic changes in glial cells and glial metabolism have long been suspected to play a key role in major depression^21,39^. GFAP is widely regarded as an established marker for astrocytic pathologies, and a decreased expression level of GFAP and reduced density of GFAP-immunoreactive astrocytes in tissue samples from various cerebral areas have been associated with major depression^11,14,40–42^. Additionally, there seems to be a difference in glial changes between early- and late-life depression, with rather subtle glial changes in elderly patients^43,44^. For the first time, the current study produces evidence of astrocytic pathology in major depression in terms of increased GFAP signals based on CSF measurements. In a recent prospective study comprising 12 patients with spinal cord injury, GFAP was found to be elevated in CSF possibly reflecting neuronal injury^45^. Accordingly, elevated levels of GFAP in CSF could also be a consequence of neuronal damage. Moreover, based on the current data, it is not possible to determine if GFAP is released by glial cells as part of a response to neuronal damage or if it is the result of glial cell loss. Further investigations are needed to connect the CSF findings to possible pathophysiological pathways that may precede GFAP release, such as the chronic mild stress response^46^.

Extensive studies on S100B in depression have been conducted in the last two decades. Elevated concentrations of S100B in serum samples have been associated with major depression, and a decrease in S100B in the course of therapy may be an indicator of therapy response, although the latter is under dispute^17,18,21,39,47^. The involvement of S100B would provide a link to inflammatory processes, as S100B can bind to the receptor for advanced glycation end-products, which is a known regulator of immunologic and inflammatory pathways^48^. In addition, in a rat model, chronic stress was found to influence S100B expression in tissue samples, thus yielding a possible connection between the stress response and depression^49^. In the current study, no significant difference in S100B levels in CSF was identified between patients with depression and controls. Therefore, the results of our study do not support the previously reported alterations in serum and animal models. However, the detected correlations between S100B levels and AQs and protein levels support the long-held hypothesis that S100B levels could be markers for disrupted blood–brain/CSF function^50^.

### Clinical and research implications

As only very limited data about the relation of GFAP in CSF to unipolar depression are currently available, this study can provide a starting point for further investigations. Given that GFAP is elevated in the CSF of patients with major depression, it may serve as an additional state or trait biomarker in depression. However, the results of previous studies to date tend to indicate a transdiagnostic phenomenon (see Table 1); thus, it is necessary to evaluate GFAP in patients with depression in combination with other alterations, such as altered cytokine levels^10^. Certainly, data indicating a possible role of GFAP in the pathogenesis or progression of major depression will add to the growing knowledge of the possible involvement of inflammatory processes in depression^51,52^. Based on our data, it is not clear if inflammatory processes are the cause of depression or if they occur as a secondary effect in patients with depression. Hence, future longitudinal studies involving CSF examinations, including the measurement of GFAP, in combination with additional immunological markers are necessary.

### Limitations

Although a large number of patients underwent CSF examinations, the current study has some relevant limitations. Because of the retrospective nature of the study, only limited psychometric data were available. In addition, the study groups were not matched for age and sex. For age, a relevant influence has been reported in previous studies. Si et al. reported a decrease in GFAP levels in postmortem brain tissue with age in 15 patients with major depression and 15 controls^12^. Therefore, age (as well as age and sex) corrected ANOVAs were performed in this study, and the results remained stable. Finally, for a sub-analysis, 1:1 matching by age and sex was performed. Again, GFAP levels were identified to be significantly elevated in patients with unipolar depression compared with matched controls. Thus, the authors concluded that the age (and sex) differences alone did not sufficiently explain the group differences. The effects of other influencing factors, such as medication, psychiatric comorbidity, or body mass index, cannot be excluded^53^. Furthermore, the presence of neurological comorbidities within the group of patients may have a confounding influence on the results. In our department, lumbar punctures are only performed in severe cases with major depression to rule out organic forms. Therefore, the results are not generalizable to all patients with unipolar depression. Because of the invasive nature of lumbar punctures, healthy controls were not recruited for ethical reasons. Additionally, this study was intended as a hypothesis-generating investigation to serve as a potential starting point for further research on GFAP and S100B levels in CSF as potential biomarkers in unipolar depression, limiting the justification for a possible control group of healthy volunteers even further. Thus, given these restrictions, patients with IIH and without psychiatric comorbidities were used as a mentally healthy control group. This approach has been established in previous studies^10,24,28^, but nevertheless, pathological processes in the IIH group may have influenced the results^54^. Preanalytical handling of the samples was primarily performed to assure measurement of the respective routine CSF parameters. Therefore, the samples were not deep-frozen directly after lumbar punctures, which would have been optimal^55–57^. However, at room temperature GFAP and S100B have been reported to be stable in CSF for up to 24 h (GFAP) and 2 days (S100B), respectively^58–60^. Storage time also did not differ between the patient and control group. Therefore, preanalytical effects are unlikely to have influenced the results.

## Conclusion

This study is one of the first to measure S100B and GFAP in the CSF of patients with unipolar depression. Elevated GFAP levels were detected; therefore, GFAP levels may serve as an additional biomarker for major depression in the future, but further investigations, including multimodal longitudinal studies, are needed to clarify the exact role and reliability of intrathecal GFAP alterations in depression.
